# Engraftment of human induced pluripotent stem cell-derived myogenic progenitors restores dystrophin in mice with duchenne muscular dystrophy

**DOI:** 10.1186/s40659-020-00288-1

**Published:** 2020-05-19

**Authors:** Ruojie He, Huan Li, Liang Wang, Yaqin Li, Yu Zhang, Menglong Chen, Yuling Zhu, Cheng Zhang

**Affiliations:** 1 Department of Neurology, The First Affiliated Hospital, Sun Yat-sen University, Guangzhou, Guangdong China; 2 Guangdong Provincial Key Laboratory of Diagnosis and Treatment of Major Neurological Diseases, National Key Clinical Department and Key Discipline of Neurology, Guangzhou, Guangdong China; 3 Department of Neurology, The Seventh Affiliated Hospital, Sun Yat-sen University, Shenzhen, Guangdong China; 4 Department of Neurology, The First Affiliated Hospital, Jinan University, Guangzhou, Guangdong China

**Keywords:** Duchenne muscular dystrophy, Human induced pluripotent stem cells, Myogenic progenitors, Muscle regeneration, Satellite cells

## Abstract

**Background:**

Duchenne muscular dystrophy (DMD) is a devastating genetic muscular disorder with no effective treatment that is caused by the loss of dystrophin. Human induced pluripotent stem cells (hiPSCs) offer a promising unlimited resource for cell-based therapies of muscular dystrophy. However, their clinical applications are hindered by inefficient myogenic differentiation, and moreover, the engraftment of non-transgene hiPSC-derived myogenic progenitors has not been examined in the mdx mouse model of DMD.

**Methods:**

We investigated the muscle regenerative potential of myogenic progenitors derived from hiPSCs in mdx mice. The hiPSCs were transfected with enhanced green fluorescent protein (EGFP) vector and defined as EGFP hiPSCs. Myogenic differentiation was performed on EGFP hiPSCs with supplementary of basic fibroblast growth factor, forskolin, 6-bromoindirubin-3′-oxime as well as horse serum. EGFP hiPSCs-derived myogenic progenitors were engrafted into mdx mice via both intramuscular and intravenous injection. The restoration of dystrophin expression, the ratio of central nuclear myofibers, and the transplanted cells-derived satellite cells were accessed after intramuscular and systemic transplantation.

**Results:**

We report that abundant myogenic progenitors can be generated from hiPSCs after treatment with these three small molecules, with consequent terminal differentiation giving rise to mature myotubes in vitro. Upon intramuscular or systemic transplantation into mdx mice, these myogenic progenitors engrafted and contributed to human-derived myofiber regeneration in host muscles, restored dystrophin expression, ameliorated pathological lesions, and seeded the satellite cell compartment in dystrophic muscles.

**Conclusions:**

This study demonstrates the muscle regeneration potential of myogenic progenitors derived from hiPSCs using non-transgenic induction methods. Engraftment of hiPSC-derived myogenic progenitors could be a potential future therapeutic strategy to treat DMD in a clinical setting.

## Background

Duchenne muscular dystrophy (DMD) (OMIM: 310200) is the most common form of inherited muscular dystrophy characterized by progressive skeletal muscle weakness and hypertrophy. It is a lethal X-linked recessive disease caused by mutations in dystrophin gene (*DMD*) (HGNC ID: 2928) [1, 2]. Dystrophin is a fundamental component of the dystrophin-associated glycoprotein complex in the sarcolemma that supports the stabilization of muscle fibers [1]. The absence of dystrophin leads to myofiber damage during muscle contraction resulting in the excessive regeneration of satellite cells and fibrotic connective tissue [3, 4]. Satellite cells are adult stem cells capable of self-renewal and myogenic differentiation. They are located between the sarcolemma and basal lamina of muscle fibers, and are distinguished by expression of paired-box transcription factor 7 (Pax7) and Pax3 [5–7]. In dystrophic muscles, the reduplicative degenerative and regenerative processes exhaust satellite cells leading to the replacement of muscle cells with resident fibrosis and adipose tissue. Notably, recent studies show that dystrophin deficiency leads to impairments in cell polarity, proliferation, and myogenic differentiation of satellite cells, and eventually impairs muscle regenerative processes [8, 9].

Currently, there are no effective therapeutic strategies for patients with DMD apart from symptomatic treatments such as the application of corticosteroids to delay disease progression [10]. Considering the evidence that muscle cell death and functional loss are strongly associated with dysfunction of satellite cells in DMD [9], transplantation of healthy satellite cells carrying a functional dystrophin gene may ameliorate muscle regeneration and self-renewal to maintain the satellite cell pool and restore dystrophin. Myoblasts served as the initial cell type candidate for replacement therapies of DMD because of the ease of accessibility in vitro [11]. Researchers have shown that transplantation of myoblasts from healthy donors partially contribute to regeneration of skeletal muscles and restore dystrophin expression in dystrophic mice as well as patients with DMD [12–15]. However, their decreased proliferative potential during culture in vitro, limited migrated capacity, and poor survival after transplantation, greatly compromise their application in clinical therapies [16]. Other adult stem cells, like bone marrow-derived side population (SP) cells [17], CD133+ cells [18, 19], mesoangioblasts [20], and adipose-derived stem cells (ADSCs) [21, 22], are reported to participate in regeneration of skeletal muscles after transplantation into dystrophic mouse or other animal models. Nevertheless, these multi-lineage stem cells display reduced proliferative potential when isolated from tissues and expanded in vitro, again limiting the required number of transplanted cells needed for therapy [6, 23, 24].

Induced pluripotent stem cells (iPSCs) share a similar self-renewal capability and pluripotency to embryonic stem cells (ESCs) [25, 26], providing an unlimited cell resource that is tailored to the specific patient for cell replacement therapy [27, 28]. In fact, autologous transplantation accompanied by decreased potential immune rejection, and transgene-free iPSC lines generated by advanced reprogramming methods without genome-integrating risk, have been showed to be more suitable for clinical cell therapy [29]. Several investigators have generated myogenic progenitors and multinucleated myotubes from mouse and human iPSCs through diverse differentiation strategies [28, 30–33]. To improve the myogenic differentiation efficiency, inducible expression of MyoD and Pax3/Pax7 was utilized in several studies to enhance myogenic induction [32, 34, 35]. Darabi et al. demonstrated that the use of ectopic expression of Pax7 in human and mouse iPSCs produces robust myogenic progenitors in vitro, which successfully engrafted to produce dystrophin-positive myofibers, resulting in functional improvements in dystrophic mice [30]. However, the safety of cell transplantation therapy using transgenic iPSC-derived myogenic progenitors limits their use for DMD therapy. Alternatively, the use of several small molecules has been shown to dramatically enhance myogenic progenitor generation derived from iPSCs. Researchers demonstrated that various combinations of small molecules like glycogen synthase kinase-3β (GSK-3β) inhibitor, forskolin, basic fibroblast growth factor (bFGF), and bone morphogenic protein (BMP) inhibitor, promote iPSCs to undergo myogenic differentiation in a high efficiency [36–39]. Nevertheless, the muscle regenerative capability of these iPSC-derived myogenic precursors induced by small molecules has not been assessed in dystrophic mouse models.

In the present study, we describe the direct derivation of skeletal myogenic progenitors from healthy human iPSCs (hiPSCs) using the three small molecules, GSK-3β inhibitor 6-bromoindirubin-3′-oxime (BIO), forskolin, and bFGF, to generate myotubes. Upon transplantation into *mdx* mice, we found that these hiPSC-derived myogenic progenitors contributed to long-term muscle regeneration and restored dystrophin expression.

## Methods

### Cell culture

The generation of hiPSCs from a healthy control donor was performed as previously described [40]. Peripheral blood mononuclear cells from healthy control donor were collected for iPSC induction. Cells were transduced with the integration-free CytoTune-iPS Sendai Reprogramming Kit (Life Technologies, Carlsbad, CA, USA), which utilizes Sendai virus particles of the four factors (*OCT4*, *SOX2*, *c*-*MYC*, and *KLF4)*. Transduced cells were plated on vitronectin-coated culture dishes and fed iPSC medium, which was replaced by StemPro 34 SFM (Life Technologies) from days 3 to 7. On day 7, the medium was replaced by feeder-free mTeSR1medium (STEMCELL Technologies, Vancouver, BC, Canada) until small colonies were formed. The growth of small colonies was maintained for another 3–4 weeks, and cell colonies were manually picked and mechanically dissociated for the first four passages. The hiPSCs were maintained on Matrigel-coated plates (BD Bioscience, Franklin Lakes, NJ, USA) with mTeSR1 medium (STEMCELL Technologies), and passaged every 4–5 days using 1 mg/mL dispase (Life Technologies). All experimental protocols including human stem cell use were approved by the Ethics Committee at the First Affiliated Hospital of Sun Yat-sen University.

### Generation of enhanced green fluorescent protein (EGFP) transgenic hiPSCs

Following the Gateway LR reaction protocol, the entry clones pUp-EF1α and pDown-EGFP were cloned into the expression vector pDes-Puro to generate an expression lentiviral vector (pLV/Final-Puro-EF1α-EGFP). The lentiviral vectors were co-transfected with packaging plasmids into 293FT cells using the X-tremeGENE Transfection Reagent (Roche, Basel, Switzerland). After 48 h, the supernatant containing lentivirus was collected, filtered through a 0.45 μm filter and concentrated by ultracentrifugation. Stable enhanced green fluorescent protein (EGFP) transgenic hiPSCs (defined as EGFP hiPSCs) were generated by lentiviral transduction after a 12 h exposure to viral particles. Next, clones of EGFP hiPSCs were selectively isolated using 1 μg/mL puromycin (Sigma-Aldrich, St. Louis, MO, USA) in culture medium for 2 weeks.

### Animals

All animal experiments were performed according to approved protocols by the Animal Care and Experimentation Committee of Sun Yat-sen University. NOD SCID (NOD.Cg-Prkdcscid/Nju) mice, C57 (C57BL/6J) mice, and *mdx* mice (C57BL/10ScSn-DMDmdx/J) were purchased from the Nanjing Biomedical Research Institute of Nanjing University (Nanjing, China). Five-to-eight-week-old NOD SCID mice were used for teratoma formation experiments, while C57 mice were used to detect dystrophin expression and 6–8-week-old male *mdx* mice were used for transplantation studies with EGFP hiPSC-derived myogenic progenitors.

### Embryoid bodies and teratoma formation

For in vitro formation of embryoid bodies (EBs), EGFP hiPSCs were digested into small clumps using 1 mg/mL dispase (Life Technologies) and plated onto low adherent petri dishes (Greiner Bio-One, Monroe, NC, USA). EBs were cultured in suspension with Essential 6 medium (Life Technologies) for 7 days with media changes every other day. At day 8, EBs were plated onto gelatin-coated plates to allow adherent culture. After an additional 7 days, the EBs were fixed and immunofluorescence assays were performed to assess three germ layer formation markers.

For teratoma formation in vivo, EGFP hiPSCs from one 6-well plate were suspended in a 1:1 mixture of Dulbecco’s Modified Eagle’s medium (DMEM) (Life Technologies) and Matrigel matrix, and injected into NOD SCID mice subcutaneously. After 8–10 weeks, teratomas were dissected and paraffin-embedded tissue sections were subjected to hematoxylin and eosin staining for germ layer histological evaluation.

### In vitro myogenic differentiation of EGFP hiPSCs

Differentiation of EGFP hiPSCs into myogenic progenitors and subsequent myotubes was performed using described protocols [36]. Briefly, hiPSCs were dissociated using 1 mg/mL dispase and plated onto low adherent petri dishes to generate EBs. The EBs were maintained in suspension culture for 7 days with a myogenic induction medium consisting of STEMDiff APEL medium (STEMCELL Technologies) supplemented with 10 ng/mL bFGF (Life Technologies), 0.5 mM BIO (Santa Cruz Biotechnology, Dallas, TX, USA), and 20 mM forskolin (Santa Cruz Biotechnology). At day 8, EBs were transferred to Matrigel-coated plates to facilitate attachment over a 3-day period. The myogenic induction medium was then switched to DMEM containing 2% horse serum (Life Technologies) for an additional 26 days of terminal myogenic differentiation with a medium change every other day.

### Transplantation of EGFP hiPSC-derived myogenic progenitors

Before intramuscular and intravenous transplantation, *mdx* mice were intraperitoneally injected with a daily dose of 20 mg/kg Busulfex (Otsuka, Tokyo, Japan) for 5 days to induce immunosuppression. The EGFP hiPSC-derived myogenic progenitors at differentiation (day 14) were used as donor cells. For intramuscular transplantation, 1 × 10^6^ cells in 50 μL phosphate-buffered saline (PBS) (Hyclone, Logan, UT, USA) were injected into the left tibialis anterior (TA) muscle, while the right TA muscle received the same volume of PBS as a control. In addition, a group of *mdx* mice received the equivalent volume of PBS in both TA muscles to serve as additional negative controls. At 4, 8, and 12 weeks after transplantation, cell-injected left TA muscles, PBS-injected right TA muscles, and both TA muscles in negative control *mdx* mice (n = 3 for each group at each time point) were biopsied and frozen using isopentane cooled in liquid nitrogen for further immunofluorescence analyses. For intravenous transplantation, 2 × 10^6^ cells in 200 μL PBS were injected into the tail vein of *mdx* mice, while a group of *mdx* mice were injected with the equivalent volume of PBS as negative controls. The TA muscles of cell-transplanted and PBS-injected *mdx* mice (n = 5 for each group) were harvested 8 weeks after transplantation for immunofluorescence studies.

### Immunofluorescence analysis

Culture cells were fixed with 4% (w/v) paraformaldehyde (Sigma-Aldrich) for 15 min, permeabilized with 0.3% (v/v) Triton X-100 (Sigma-Aldrich) in PBS for 15 min, and then blocked with 5% (w/v) bovine serum albumin (BSA) (Sigma-Aldrich) in PBS for 1 h at room temperature. Next, cell were incubated with primary antibodies diluted in 5% (w/v) BSA overnight at 4 °C. The following primary antibodies were used: OCT4 (1:100, Santa Cruz Biotechnology), SOX2 (1:200, Abcam, Cambridge, UK), TRA-1-60 (1:100, Merck Millipore, Billerica, MA, USA), TRA-1-81 (1:100, Merck Millipore), Nestin (1:200, Abcam), alpha 1 Fetoprotein (AFP) (1:100, Abcam), alpha smooth muscle actin (αSMA) (1:200, Abcam), PAX7 (1:100, Abcam), MYF5 (1:100, Abcam), Desmin (1:100, Abcam), MF20 for myosin heavy chain (MHC) (1:100, Developmental Studies Hybridoma Bank, University of Iowa, Iowa City, IA, USA), and DYS1 (1:100, Leica Biosystems, Wetzlar, Germany). After primary antibody incubation, cells were washed three times with PBS and incubated at room temperature for 1 h with the corresponding Alexa Fluor 555-conjugated anti-mouse or anti-rabbit secondary antibody (1:1000, Cell Signaling Technology, Danvers, MA, USA). Nuclei were counter-stained with 4′,6-diamidino-2-phenylindole (DAPI) (Sigma-Aldrich). Images were captured using an IX71 fluorescence microscope (Olympus, Tokyo, Japan).

For tissue sections, serial 10-μm-thick cryosections of muscle tissues were collected, fixed with cold acetone for 10 min at 4 °C, and blocked with 10% (v/v) fetal bovine serum (FBS) and 2% horse serum for 1 h at room temperature. Sections were then incubated with primary antibodies against DYS1 (1:100, Leica Biosystems, Wetzlar, Germany), human spectrin (1:100, Abcam), GFP (1:100, Abcam), and Pax7 (1:100, Abcam) overnight at 4 °C. Tissue sections were washed three times with PBS and stained with Alexa Fluor 555-conjugated or Alexa Fluor 488-conjugated secondary antibodies (1:1000, Cell Signaling Technology) for 1 h at room temperature. Nuclei were counter-stained with DAPI. Images were captured and analyzed using a DS-Ri2 fluorescence microscope (Nikon, Tokyo, Japan).

### Western blot analysis

Cells cultured on dishes were washed using cold PBS and lysed with RIPA Lysis Buffer (Thermo Fisher Scientific, Waltham, MA, USA) in the presence of protease and phosphatase inhibitors (Thermo Fisher Scientific) on ice for 30 min. For muscle tissue preparation, samples were disrupted with a Tissue Lyser II (Qiagen, Hilden, Germany) using RIPA Lysis Buffer supplemented with protease and phosphatase inhibitors as well as 0.5 M EDTA (Invitrogen), followed by a 30-min incubation on ice with intermittent vortexing. The lysates from cultured cells or muscle tissues were ultrasonicated, centrifuged at 12,000×*g* for 30 min at 4 °C, and the soluble protein supernatant was collected. The total protein concentration was measured using a Pierce BCA Assay Kit (Thermo Fisher Scientific) according to the manufacturer’s protocol. Samples containing identical amounts of protein (30 μg) were loaded and run in 10% (w/v) sodium dodecyl sulfate polyacrylamide (SDS-PAGE) gels (Invitrogen).For detecting dystrophin, 6% SDS-PAGE gels were used. Then the proteins were transferred to 0.45 μm pore-sized polyvinylidene difluoride (PVDF) membranes (Merck Millipore). The membranes were blocked with 5% (w/v) BSA in Tris-buffered saline with Tween 20 (TBS/T) (Sigma-Aldrich) for 1 h at room temperature, and incubated overnight at 4 °C with primary antibodies as follows: PAX7 (1:500, Developmental Studies Hybridoma Bank), MYF5 (1:500, Abcam), Desmin (1:1000, Abcam), MF20 (1:500, Developmental Studies Hybridoma Bank), DYS1 (1:200, Leica Biosystems, Wetzlar, Germany), GAPDH (1:1000, Cell Signaling Technology), and β-Tubulin (1:1000, Cell Signaling Technology). Membranes were washed three times with TBS/T and incubated with anti-rabbit or anti-mouse HRP-linked secondary antibodies (1:1000, Cell Signaling Technology) for 1 h at room temperature. Proteins were visualized using the immobilon western chemiluminescent HRP substrate (Merck Millipore) and an ImageQuant LAS 4000 detection system (GE Healthcare Life Sciences, Chicago, IL, USA). Protein expression levels were normalized to GAPDH or β-Tubulin and quantified using Image J software.

### Hematoxylin and eosin staining of muscle tissue sections

Hematoxylin and eosin (H&E) staining was performed to detect the pathological lesions of muscles. Serial 10-μm-thick cryosections of muscle tissues were collected, fixed with cold acetone for 10 min at 4 °C. Cryosections were stained with the haematoxylin for nuclei staining for 4 min, rinsed in running tap water, differentiated with 1% acid alcohol for 2 s, rinsed in running tap water again, and then rinsed in Scott’s tap water substitute for blueing up for 20 s and rinsed in running tap water. Cryosections were stained with eosin for 2 min, dehydrated, cleared, and mounted with neutral resins.

### Statistics

All data are presented as mean ± standard error of the mean (SEM) and statistically analyzed by GraphPad Prism. Differences between two groups of samples were assessed using two-tailed Student’s t test while the one-way analysis of variance (ANOVA) was used for multiple comparison between groups. *P* < 0.05 was considered statistically significant.

## Results

### Retention of pluripotent characterization on EGFP hiPSCs

**Fig. 1:**
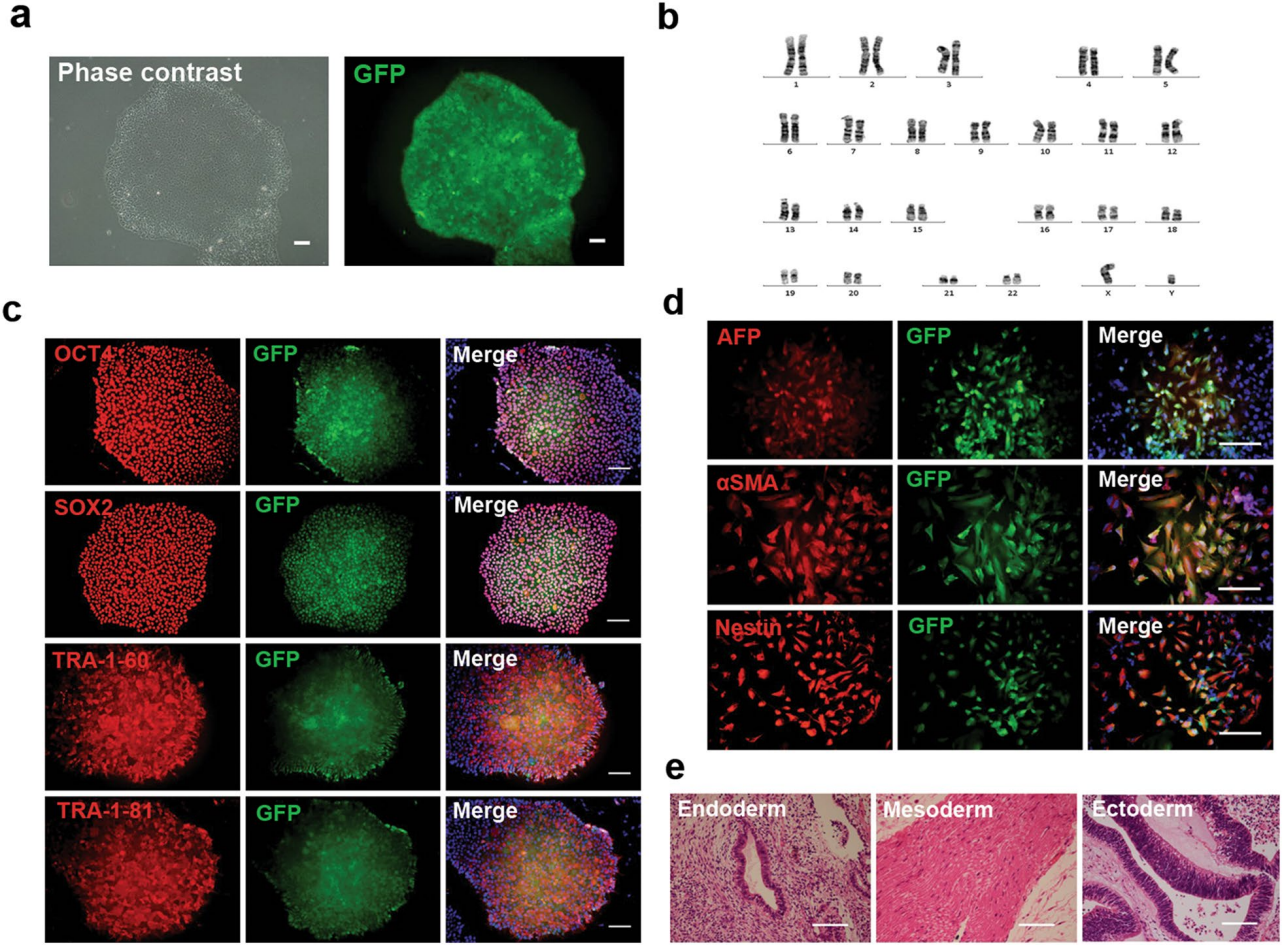
Generation and characterization of EGFP hiPSCs. **a** Phase contrast and fluorescence micrographs showed morphology and GFP expression in hiPSCs transduced with the EGFP expression vector. **b** EGFP hiPSCs retained normal karyotype. **c** Immunofluorescence analyses of the pluripotent markers, OCT4, SOX2, TRA-1-60, and TRA-1-81, in EGFP hiPSCs. **d** EGFP hiPSCs differentiated into three germ layers via EB formation, as indicated by the expression of AFP, αSMA, and Nestin in immunofluorescence staining. **e** Hematoxylin and eosin (H&E) staining of teratoma formed from EGFP hiPSCs showed endoderm-derived glandular epithelium, mesoderm-derived smooth muscle, and ectoderm-derived neural tube. Scale bars = 100 μm

To facilitate tracking of transplanted hiPSC-derived myogenic progenitors, a lentivirus vector overexpressing green fluorescent protein (GFP) was constructed (Additional file 1: Figure S1) and transfected into healthy control hiPSCs. After puromycin selection for 14 days, the majority of cells (> 90%) were infected, as shown by GFP expression (Fig. 1a). GFP-positive colonies were selected, maintained, and used for further experiments. The morphology of EGFP hiPSCs resembled that of untransfected hiPSCs (Fig. 1a) and could be passaged as usual with normal karyotype (Fig. 1b). To further test whether EGFP hiPSCs retained pluripotent characterization, analysis of pluripotency markers was performed and EGFP hiPSCs were induced to differentiate into three germ layers in vitro and in vivo. Immunocytochemistry studies showed that EGFP hiPSCs expressed pluripotency markers, such as OCT4, SOX2, TRA-1-60, and TRA-1-81 (Fig. 1c). Accordingly, the negative control experiments of immunocytochemistry on iPSCs showed that no unspecific immunofluorescence was detected on secondary antibodies without primary antibody (Additional file 1: Figure S2). Similar to untransfected hiPSCs, EGFP hiPSCs could form EBs containing three germ layers in vitro, and expressed specific markers of endoderm (AFP), mesoderm (αSMA), and ectoderm (nestin) (Fig. 1d). Furthermore, 2 months after EGFP hiPSCs were subcutaneously injected into NOD SCID mice, teratoma formation was detected and analyzed. Our histological examination revealed that teratomas contained tissues of three germ layers, including glandular epithelium (endoderm), smooth muscle (mesoderm), and neural tube (ectoderm) (Fig. 1e). These results demonstrate that EGFP hiPSCs harbored the pluripotent stem cell features of self-renewal and multipotential differentiation.

### Myogenic differentiation from EGFP hiPSCs in vitro

**Fig. 2:**
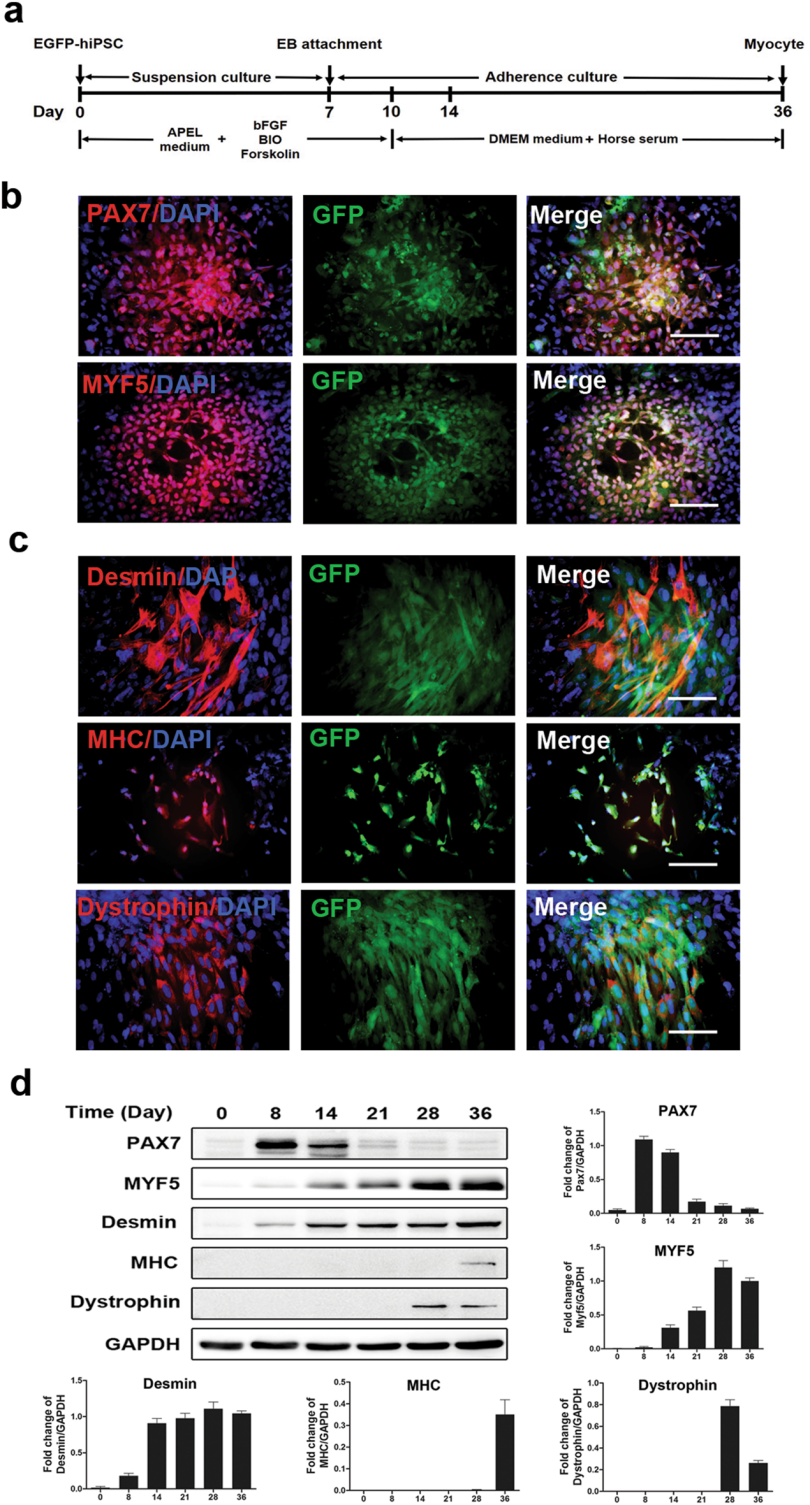
Myogenic differentiation of EGFP hiPSCs in vitro. **a** Experimental scheme for myogenic progenitor induction from EGFP hiPSCs followed by myogenic maturation. **b** Immunofluorescence analysis indicated expression of the early myogenic markers PAX7 and MYF5 at differentiation day 14. **c** Late myogenic markers, Desmin, MHC, and dystrophin were detected by on day 36 via immunofluorescence analysis. **d** Western blot analysis of myogenic markers at different time points during myogenic differentiation of EGFP hiPSCs (All data are presented as the mean ± SEM, n = 3 independent differentiation experiments). Scale bars = 100 μm

In an earlier study, we showed that engraftable myogenic progenitors could be effectively generated from mouse ADSCs with supplementation of the small molecules including BIO, forskolin and bFGF [22]. The differentiation procedure to obtain robust myogenic progenitors and the terminal myofibers from EGFP hiPSCswas showed in Fig. 2a and Additional file 1: Figure S3. Immunofluorescence analyses revealed robust expression of the early myogenic markers PAX7 and MYF5, as well as Myogenin and MyoD1 at differentiation day 14 (Fig. 2b and Additional file 1: Figure S4). At differentiation day 36, the expression of late skeletal muscle markers desmin, MHC, and dystrophin were detected, indicating maturation of myogenic progenitors within the culture (Fig. 2c). Moreover, we evaluated the expression of each myogenic marker at different time points during myogenic differentiation using western blot analyses. The results demonstrated that the amount of PAX7 peaked on day 8 and then gradually decreased (Fig. 2d). We also found that the amount of MYF5 and desmin kept increasing during differentiation process, while that of MHC and dystrophin increased from day 36 and day 28, respectively (Fig. 2d). Satellite cells are regarded as the preferable cell type for cell transplantation therapy because of their self-renewal and muscle regeneration potential, and Pax7 is the crucial myogenic transcriptional factor specifically expressed in satellite cells. Therefore, myogenic differentiation day 8 was chosen as the most suitable timepoint for transplantation of EGFP hiPSC-derived myogenic progenitors into *mdx* mice because of the peak expression level of PAX7.

### Intramuscular transplantation of EGFP hiPSC-derived myogenic progenitors restore dystrophin expression in *mdx* mice

**Fig. 3:**
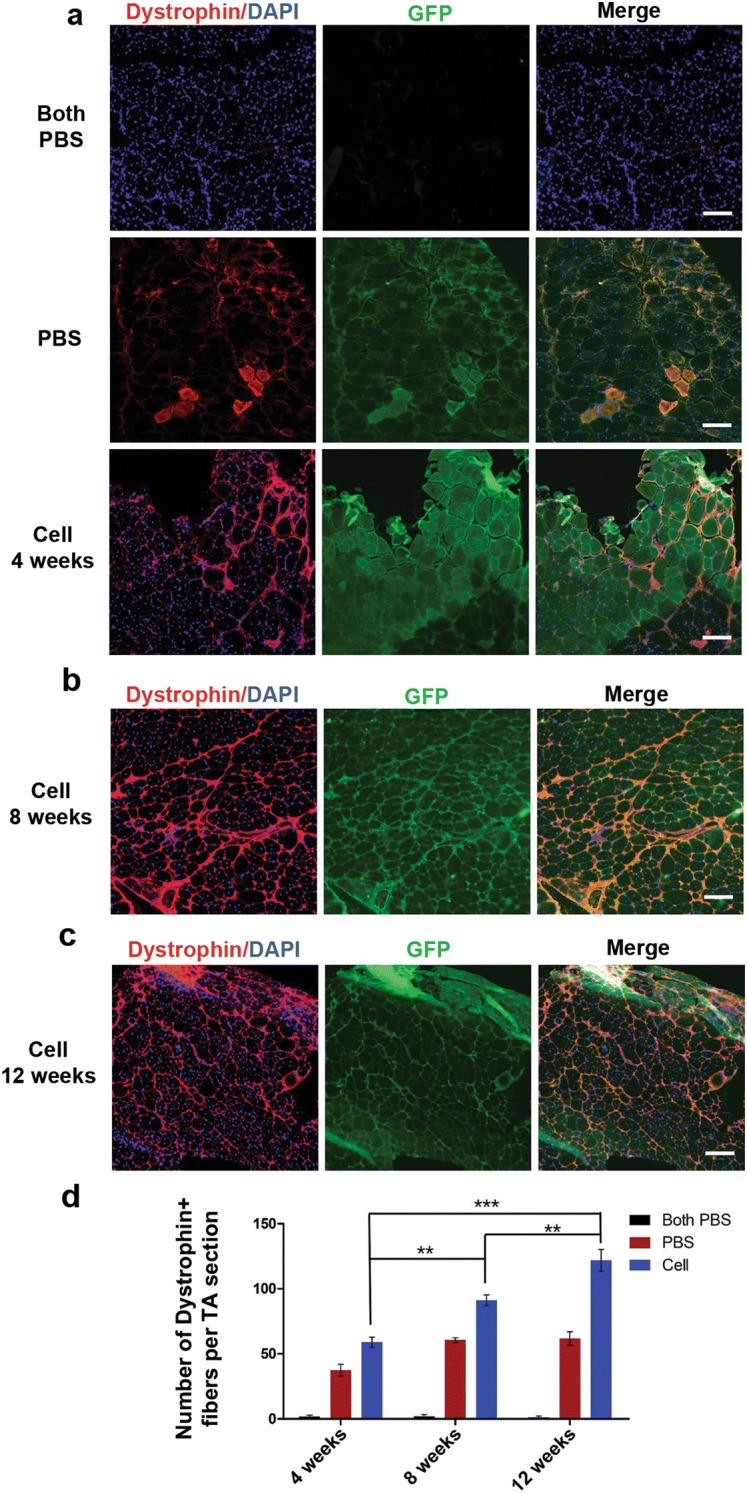
Restoration of dystrophin expression in mdx mice after intramuscular transplantation of EGFP hiPSC-derived myogenic progenitors. **a** Immunofluorescence analysis showed no expression of dystrophin (red) and GFP (green) in TA muscles of negative control mdx mice (upper panels), while dystrophin and GFP double expression in PBS-injected right TA muscles (middle panels) and cell-transplanted left TA muscles (lower panels) at 4 weeks after transplantation. **b** Immunofluorescence analyses showed dystrophin and GFP double positive myofibers in cell-transplanted left TA muscles at 8 weeks after transplantation. **c** Immunofluorescence analyses showed dystrophin and GFP double positive myofibers in cell-transplanted left TA muscles at 12 weeks after transplantation. **d** Quantitative analysis demonstrated the number of dystrophin-positive myofibers for each group at 4, 8, and 12 weeks after transplantation. Data are presented as the mean ± SEM (5 random sections for each muscle were examined). **P < 0.01, ***P < 0.001. Scale bars = 400 μm

To investigate the muscle regenerative potential of EGFP hiPSC-derived myogenic progenitors in vivo, cells were transplanted into *mdx* mice, a DMD mouse model characterized by lacking dystrophin expression. We confirmed that the *mdx* mice used as recipients were deficient in dystrophin expression compared to that found in the muscles of normal C57 mice (Data not shown). At 4 weeks after transplantation, dystrophin-positive myofibers regenerated from EGFP hiPSC-derived myogenic progenitors (expressing GFP) were detected in cell-transplanted left TA muscles as well as PBS-injected right TA muscles, while just few reversed myofibers was observed in *mdx* mice that received PBS in both TA muscles (Fig. 3a). Quantitative analysis showed that the number of dystrophin-positive myofibers per TA section in negative control muscles, PBS-injected muscles, and cell-injected muscles were 1.80 ± 1.11, 37.40 ± 4.45, 59.00 ± 3.99, respectively (Fig. 3d). In addition, 8 weeks after transplantation, resemble muscle engraftment as 4 weeks was observed in both cell-transplanted and PBS-injected TA muscles as demonstrated by the number of myofibers co-expressing GFP and dystrophin (Fig. 3b). Quantitative analysis showed that the number of dystrophin-positive myofibers per TA section in negative control muscles, PBS-injected muscles, and cell-injected muscles were 2.00 ± 1.30, 60.60 ± 1.86, 91.20 ± 4.72, respectively (Fig. 3d).

Long-term engraftment and dystrophin protein restoration have been observed in several studies by transplanting mouse ADSC-derived myogenic progenitors [22], human mesenchymal stem cells (MSCs) [41], or mouse bone marrow-derived cells [42] into *mdx* mice, while long-term muscle incorporation after transplantation of hiPSC/hESC-derived cells into *mdx* mice has not been previously investigated. In the present study, to further test whether the myogenic progenitors differentiated from EGFP hiPSCs could contribute to long-term engraftment, we prolonged the observation duration to 12 weeks after intramuscular transplantation. Notably, we identified a considerable number of dystrophin-positive myofibers derived from GFP-expressing donor cells in the left TA muscles that received cells (121.80 ± 8.35) (Fig. 3c and Additional file 1: Figure S5) as well as the contralateral TA muscles that received PBS (61.80 ± 5.23) (Data not shown). It is noteworthy that quantitative analysis dystrophin-positive myofibers showed the number of dystrophin-positive myofibers in cell-injected muscles at 8 weeks after transplantation was higher than that at 4 weeks (P < 0.01), and the number of dystrophin-positive myofibers at 12 weeks after transplantation was higher than that at 8 weeks (P < 0.01) (Fig. 3d). In the *mdx* mice received cell injection, we detected the expression of human spectrin, indicating human cell derived myofibers (Additional file 1: Figure S6). Overall, our results strongly indicated that intramuscular transplantation of EGFP hiPSC-derived myogenic progenitors were competent to engraft into the muscles of *mdx* mice and restore dystrophin expression. Meanwhile, the number of dystrophin positive myofibers in cell-injected muscles increased along with the engrafted time went by.

### EGFP hiPSC-derived myogenic progenitors engrafted into the muscles of *mdx* mice via systemic transplantation

**Fig. 4:**
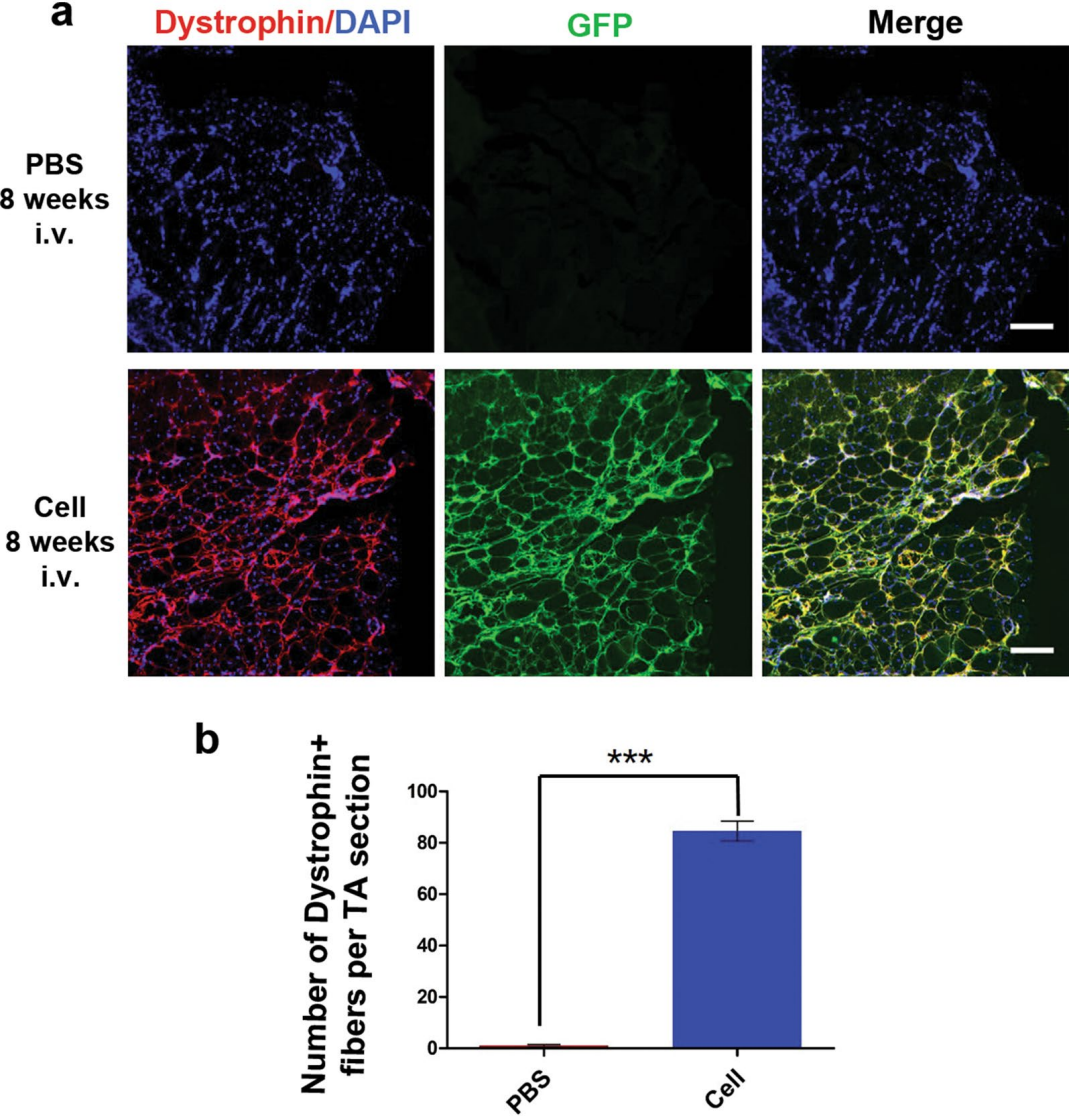
Systemic delivery of EGFP hiPSC-derived myogenic progenitors engrafted in muscles of mdx mice. **a** Immunofluorescence assays showed no dystrophin and GFP expression was observed in the muscles of negative control mdx mice (upper panel), while the expression of dystrophin (red) and GFP (green) in the intravenously-injected TA muscles (lower panel) was detected after 8 weeks of transplantation. **b** Quantitative analysis of dystrophin-positive myofibers in the muscles of cell-transplanted and PBS-injected mdx mice. Data are presented as the mean ± SEM (5 random sections for each muscle were examined). ***P < 0.001. Scale bars = 400 μm

Systemic transplantation of iPSC-derived myogenic progenitors has not been previously performed in dystrophic mouse models. To determine whether EGFP hiPSC-derived myogenic progenitors transplanted into *mdx* mice through tail vein injection are capable to fuse into host myofibers and contribute to dystrophin restoration, cells at myogenic differentiation day 8 were injected into *mdx* mice via tail vein, while a group of *mdx* mice received the same volume of PBS as controls. At 8 weeks after intravenous transplantation, all *mdx* mice received cells or PBS were alive and showed no functional changes on gait and rotarod test prior to sample collection (data not shown). No tumor formation was found in *mdx* mice received cells transplantation (data not shown). We found that few detectable dystrophin-positive fibers without GFP was observed in TA muscles. In contrast, significant engraftment of transplanted cells was detected in TA muscles from *mdx* mice with systemic transplantation of EGFP hiPSC-derived myogenic progenitors, as demonstrated by the presence of robust GFP and dystrophin double-positive myofibers (Fig. 4a and Additional file 1: Figure S7). Quantitative analysis revealed that dystrophin-positive myofibers per TA section in cell-transplanted *mdx* mice was significantly higher than that in negative control *mdx* mice (84.60 ± 3.84 vs. 1.00 ± 0.55 respectively, P < 0.001) (Fig. 4b). No GFP positive cells were detected in other organs like heart and liver (data not shown). Our results indicated that myogenic progenitors transplanted by intravenous injection can be recruited to engraft into dystrophin-deficient muscles and fuse to regenerate donor cell-derived myofibers, thereby resulting in dystrophin restoration in vivo.

### EGFP hiPSC-derived myogenic progenitors treatment ameliorate pathological lesions in *mdx* mice

**Fig. 5:**
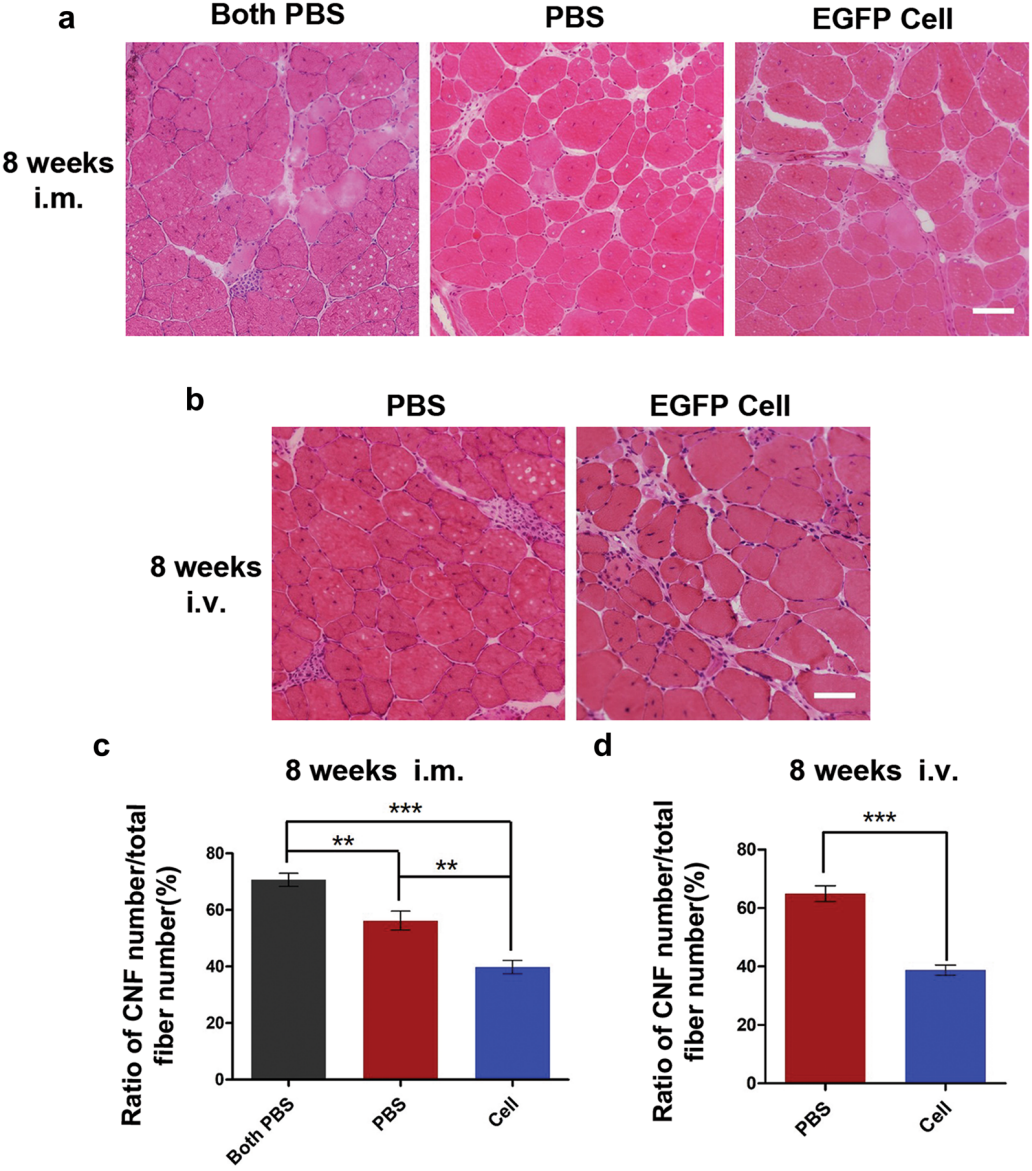
Treatment of EGFP hiPSC-derived myogenic progenitors reduced the ratio of central nuclei myofibers (CNFs) in mdx mice. **a** H&E staining showed representative images of TA muscles in negative control mdx mice (left), PBS-injected right TA muscles (middle), and cell-transplanted left TA muscles (right) at 8 weeks after intramuscular transplantation. **b** H&E staining showed representative images of TA muscles in mdx mice received PBS (left) and cells (right) at 8 weeks after intravenous transplantation. **c** Quantitative analysis indicated the percentage of CNFs for each group at 8 weeks after intramuscular transplantation. **d** Quantitative analysis indicated the percentage of CNFs for each group at 8 weeks after intravenous transplantation. 5 random sections for each muscle were examined. **P < 0.01, ***P < 0.001. Scale bars = 400 μm

An increased number of central nuclear myofibers (CNFs) is one of remarkable pathological characteristics in the muscles of DMD. In the intramuscular transplantation experiment, 8 weeks after cell transplantation, H&E staining analysis revealed that the ratio of CNFs in TA muscles of negative control *mdx* mice was up to 70.64% ± 2.35%, while the percentage of CNFs in TA muscles received cell-injection and contralateral PBS-injected TA muscles decreased significantly (39.82% ± 2.38 and 56.20% ± 3.36%, respectively) (Fig. 5a, c). For systemic transplantation therapy, H&E staining analysis indicated that the percentage of CNFs in TA muscles of *mdx* mice with intravenous cell injection (38.75% ± 1.74%) decreased compared to that in TA muscles of *mdx* mice with PBS injection (64.90% ± 2.72%) (Fig. 5b, d). As a matter of concern on therapeutic application of stem cell transplantation, we also evaluated the muscle regenerative potential of hiPSCs-derived myogenic progenitors without transfecting EGFP. Similarly, at 8 weeks after systemic transplantation, H&E staining analysis indicated that the ratio of CNFs number in TA muscles of *mdx* mice with intravenous cell injection (23.07% ± 1.68%) decreased significantly compared to that of *mdx* mice with injecting PBS intravenously (51.33% ± 3.13%) (Additional file 1: Figure S8).

### EGFP hiPSC-derived myogenic progenitors contribute to the satellite cell compartment

**Fig. 6:**
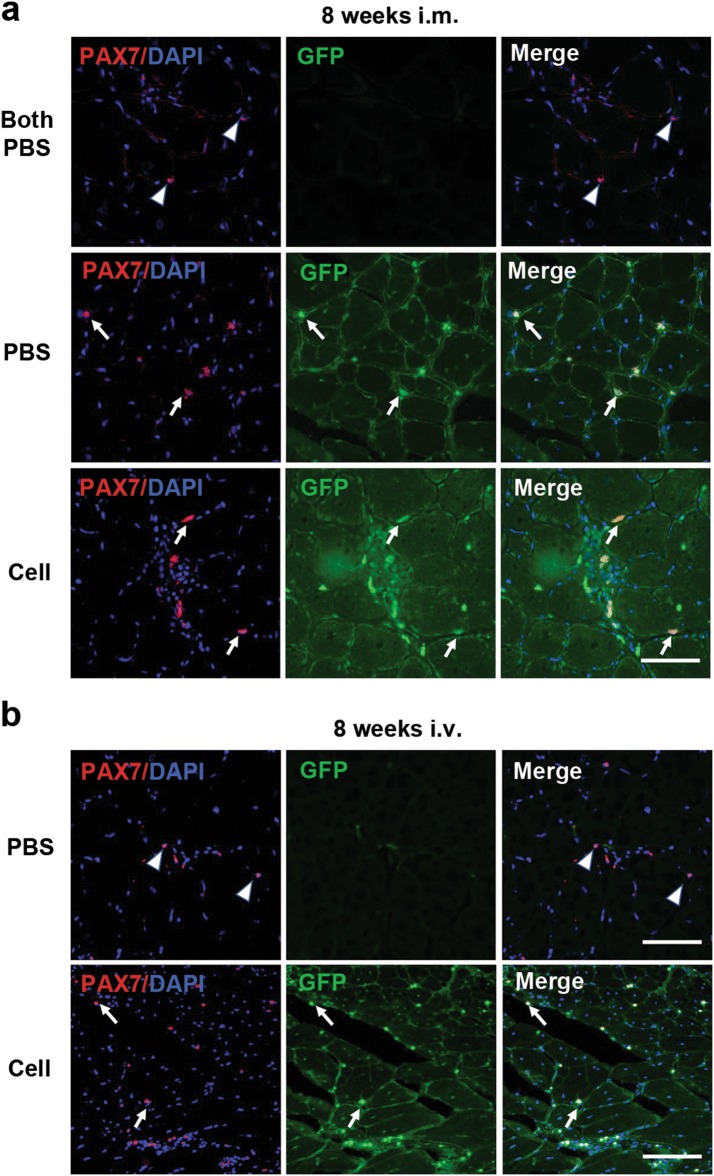
Transplantation of EGFP hiPSC-derived myogenic progenitors replenished the satellite cell compartment. **a** For intramuscular transplantation, immunofluorescence analysis showed PAX7-positive but GFP-negative cells (white arrowheads) in negative control mdx mice (upper panels), PAX7 and GFP double-positive cells (white arrows) in cell-transplanted left TA muscles (lower panel) and their counterparts that received PBS (middle panel). **b** For intravenous transplantation, immunofluorescence analysis showed PAX7-positive but GFP-negative cells (white arrowheads) in TA muscles of mdx mice received PBS (upper panels), whereas PAX7 and GFP double-positive cells (white arrows) in TA muscles of mdx mice received cells (upper panels). Scale bars = 400 μm

To examine whether EGFP hiPSC-derived myogenic progenitors are endowed with the ability to undergo self-renewal and contribute to the satellite cell compartment, immunofluorescence analyses of the satellite cell specific marker PAX7 were performed. At 8 weeks after intramuscular transplantation, we found that GFP and PAX7 double-positive cells were observed around the engrafted myofibers in TA muscles that received cells as well as their counterparts injected with PBS in the same *mdx* mice (Fig. 6a), a finding which suggests donor cell-derived satellite cells can replenish the satellite cell pool in host muscles. In contrast, no satellite cell engraftment was detected in negative control *mdx* mice that received PBS in both TA muscles, as demonstrated by the sole presence of host-originated satellite cells, which are PAX7-positive cells lacking GFP expression (Fig. 6a). Similarly, at 8 weeks after intravenous transplantation, PAX7 and GFP double positive nuclei were detected in TA muscles of *mdx* mice received cell injection but not found in *mdx* mice injected with PBS (Fig. 6b). Our results demonstrated that transplantation of EGFP hiPSC-derived myogenic progenitors contribute to seed the satellite cell pool.

## Discussion

DMD is characterized by deficient muscular dystrophin protein and repeated rounds of skeletal muscle degeneration and regeneration leading to the exhaustion of myogenic stem cells [43]. There are no effective treatments for DMD patients to date, and pharmacological treatments such as glucocorticoid administration are insufficient to improve the disease phenotype and reverse its devastating prognosis [11]. More effective potential therapeutic strategies, including cell-based therapy, gene therapy as well as exon skipping, have been evaluated on DMD animal models and have undergone clinical trials [6]. For successful stem cell-based therapies, transplanted cells expressing functional dystrophin protein should be able to fuse with recipient myofibers, participate in muscle regeneration, and ideally replenish satellite cell pool to support long-term engraftment [44]. In addition to myoblasts or satellite cells which are initially utilized for transplantation in *mdx* mice and DMD patients, other adult stem cells including mesoangioblasts, CD133+ cells, and bone marrow-derived cells have been shown to exert muscle regeneration potential in vivo [27]. Although transplantations using these adult stem cells show apparent engraftment in *mdx* mice, their clinical application is hindered by their limited ability for expansion ex vivo, poor survival, and reduced migration after transplantation [11, 24, 27]. Notably, ESCs/iPSCs overcome many of these disadvantages due to their virtually unlimited number of donor derived cells for muscle repair. Furthermore, ESC/iPSC-derived myogenic progenitors exhibit higher proliferative and migration ability than those derived from adult stem cell [44, 45]. However, most studies using ESCs/iPSCs to generate myogenic progenitors rely on overexpression of transcription factors MyoD or Pax7 before transplantation, with an inherent risk of insertional mutagenesis rendering these cells unsuitable for clinical use [46].

In the present study, we generated healthy donor-derived hiPSCs expressing EGFP in order to track donor cells in vivo and demonstrated that they efficiently differentiate into myogenic progenitors in a serum-free culture system with the induction of small molecules. Myogenic differentiation from hiPSCs using the defined medium containing BIO, bFGF, and forskolin had been reported in a previous study, but the muscle regeneration potential of these hiPSC-derived myogenic progenitors has not been evaluated in *mdx* mice [36]. Our previous work showed that myogenic progenitors differentiated from mouse ADSCs with the treatment of these three molecules could contribute to long-term engraftment in the muscles of *mdx* mice [22]. Interestingly, in the present study, the peak point of Pax7 expression (day8) during myogenic differentiation is earlier than previous work (day14), suggesting distinct myogenic induction efficiency between cell types or cell lines. Similarly, we demonstrated that EGFP hiPSC-derived myogenic progenitors could process maturation in vitro and differentiate into myotubes expressing the late skeletal muscle markers desmin, MHC, and dystrophin, with the absence of Pax7 expression.

Our in vivo cell transplantation studies demonstrated successful incorporation of EGFP hiPSC-derived myogenic progenitors in intramuscular injected *mdx* mice pre-treated with immunosuppressant Busulfex. Herein, we detected engraftment contributions to myofiber regeneration and restoration of dystrophin expression in recipient muscles, and the donor cell-derived myofiber engraftment was observed up to 12 weeks. It is notable that other studies point out that the embryonic origin of ESC/iPSC-derived progenitors makes them more suitable for cell replacement therapy due to their capacity of expansion and migration [45]. This may explain why we detected donor engraftments in the PBS-injected TA muscles that were contralateral to the cell-injected muscles. While this may raise safety concerns, we were unable to detect any teratomas in any cell injected mice. The chemokine receptor CXCR4 is highly expressed in satellite cells as well as cultured myogenic progenitors, while its ligand CXCL12 is overexpressed in dystrophic muscles [47]. CXCL12/CXCR4 signaling plays an essential role in migration of muscle cells and efficient muscle regeneration [48–50]. This suggests a circulatory migratory recruitment mechanism that explains the contralateral detection in PBS-injected muscles that merits further studies.

Because whole-body muscles are affected in DMD patients, the desirable engrafted cells should be able to cross the blood barrier to allow more general systemic delivery. Myogenic progenitors obtained from mouse iPSCs [51], CD133+ cells [18], and SP cells [52] as well as mesoangioblasts [20] have shown significant myofiber engraftment following intravenous or intra-arterial transplantation, making systemic delivery a promising transplanting strategy for cell-based therapy in future clinical application. Similarly our previous study showed that intravenous transplantation of myogenic progenitors derived from ADSCs using BIO, bFGF and forskolin cocktail induction protocol resulted in considerable muscle engraftment in *mdx* mice [22]. The present study expanded these observations by systemically delivering EGFP hiPSC-derived myogenic progenitors. These successfully contributed to significant muscle regeneration and restored dystrophin expression, as shown by GFP/dystrophin double positive myofibers in cell-injected *mdx* mice, suggesting that transplanted cells in circulation were recruited and inhabited within deficient host muscles [52]. Importantly, no tumor formation was observed during transplantation, which confirmed the safety of systemic delivery as a potential therapeutic strategy for clinical use. Furthermore, as a matter of increasing concern in modern clinical therapeutics, it is of great value to assess muscle regeneration potential of hiPSC-derived myogenic progenitors in female animal models. When transplanting male hiPSC-derived myogenic progenitors into female *mdx* mice, the detection of Y chromosome in the muscle of female receptor could be utilized to evaluate the donor cell-derived myofiber engraftment. Further studies are needed to investigate the feasibility of transplanting male-derived stem cells into female animal models. It is demonstrated that the intrinsic satellite cell dysfunction plays an essential role in pathological progression of DMD [9]. In *mdx* muscles, dystrophin deficiency impairs polarity and subsequent asymmetric cell division of satellite cells resulting in reduced myogenic potential and loss of muscle regenerative capacity [8, 53]. Along these lines, it is conceivable that engraftment of functional satellite-like cells would contribute to muscle regeneration by replenishing satellite cell pool in *mdx* mice. Furthermore, restoration of dystrophin protein transplanted cells may facilitate intrinsic satellite cells to undergo myogenic commitment and enhance muscle repair. In the present study, we showed that EGFP hiPSC-derived myogenic progenitors using non-transgenic induction protocol incorporated into satellite cell compartment, likely improving crucial and sustainable myofiber regeneration and restoration of dystrophin. Supporting these observations, consistent dystrophin expression was observed in PAX7 and GFP double positive satellite transplanted cells. The presence of donor cell-derived myofibers and satellite cells support that transplanted myogenic progenitors migrate into circulatory system, and are recruited by dystrophic muscles.

## Conclusion

We generated healthy donor hiPSCs-derived transgene-free and serum-free myogenic progenitors which were capable of engrafting into host myofibers, participate in muscle regeneration, restore dystrophin expression, and replenish satellite cell niche following intramuscular transplantation into *mdx* mice. Furthermore, we demonstrated that these myogenic progenitors can also be delivered by intravenous transplantation to contribute to muscle engraftment, with significant implications for the development of cell-based therapeutics in DMD patients.

## Supplementary information

**Additional file 1: Figure S1.** Schematic diagram of construction of the lentiviral vector pLV/Final-Puro-EF1α-EGFP. The entry vectors, pUp-EF1α and pDown-EGFP were generated and recombined into the pDes-Puro vector utilizing the recognized LR reaction protocol.

**Additional file 2: Figure S2.** The negative control of immunocytochemistry analysis on iPSCs. No unspecific immunofluorescence was detected on Alexa Fluor 555-conjugated anti-mouse secondary antibodies (upper panle) and Alexa Fluor 555-conjugated anti-rabbit secondary antibodies (lower panle) without primary antibody.

**Additional file 3: Figure S3.** Representative phase contrast (A-E) and matching GFP fluorescence (F-J) images during myogenic differentiation at several time points. (A, F) EGFP hiPSC colonies at day 0. (B, G) EBs in suspension culture at day 4. (C, H) EB-derived monolayer cells at day 10 after plating EBs on Matrigel-coated plates. (D, I) proliferating monolayer of myogenic progenitors at day 14. (E, J) spindle-shaped myocytes at day 36. Scale bars = 100 μm.

**Additional file 4: Figure S4.** Immunofluorescence analysis indicated expression of myogenic markers Myogenin and MyoD1 at differentiation day 14. Scale bars = 100 μm.

**Additional file 5: Figure S5.** Immunofluorescence analysis showed no expression of dystrophin (red) and GFP (green) in TA muscles of negative control mdx mice (upper panels), while dystrophin and GFP double expression in PBS-injected right TA muscles (middle panels) and cell-transplanted left TA muscles (lower panels) at 12 weeks after transplantation. Scale bars = 200 μm.

**Additional file 6: Figure S6.** At 12 weeks after transplantation, immunofluorescence assays showed the expression of human spectrin in the cell-transplanted left TA muscles as well as contralateral muscles. Western blot analysis confirmed the expression of human spectrin. Scale bars = 400 μm.

**Additional file 7: Figure S7.** Immunofluorescence assays showed no dystrophin and GFP expression was observed in the muscles of negative control mdx mice (upper panel), while the expression of dystrophin (red) and GFP (green) in the intravenously-injected TA muscles (lower panel) was detected after 8 weeks of transplantation. Scale bars = 200 μm.

**Additional file 8: Figure S8.** Systemic transplantation of hiPSC-derived myogenic progenitors without transfecting EGFP reduced the ratio of central nuclei myofibers (CNFs) in mdx mice. (A) H&E staining showed representative images of TA muscles in mdx mice received PBS (left) and cells (right) at 8 weeks after intravenous transplantation. (B) Quantitative analysis indicated the percentage of CNFs for each group at 8 weeks after intravenous transplantation. 5 random sections for each muscle were examined. **P < 0.01, Scale bars = 400 μm.

## Data Availability

All data generated or analysed during this study are included in this published article.

## Abbreviations

DMD: Duchenne muscular dystrophy
iPSCs: Induced pluripotent stem cells
EGFP: Enhanced green fluorescent protein
PAX7: Paired-box transcription factor 7
ADSCs: Adipose-derived stem cells
ESCs: Embryonic stem cells
GSK-3β: Glycogen synthase kinase-3β
bFGF: Basic fibroblast growth factor
BMP: Bone morphogenic protein
BIO: 6-Bromoindirubin-3′-oxime
EBs: Embryoid bodies
DMEM: Dulbecco’s Modified Eagle’s medium
MHC: Myosin heavy chain
TA: Tibialis anterior
DAPI: 4′,6-Diamidino-2-phenylindole
CNFs: Central nuclear myofibers
