# Supplementary figures and images for: Engraftment of human induced pluripotent stem cell-derived myogenic progenitors restores dystrophin in mice with duchenne muscular dystrophy
Source: Biol Res. 2020 May 19;53:22. doi: 10.1186/s40659-020-00288-1 (PMC7238630; doi:10.1186/s40659-020-00288-1)

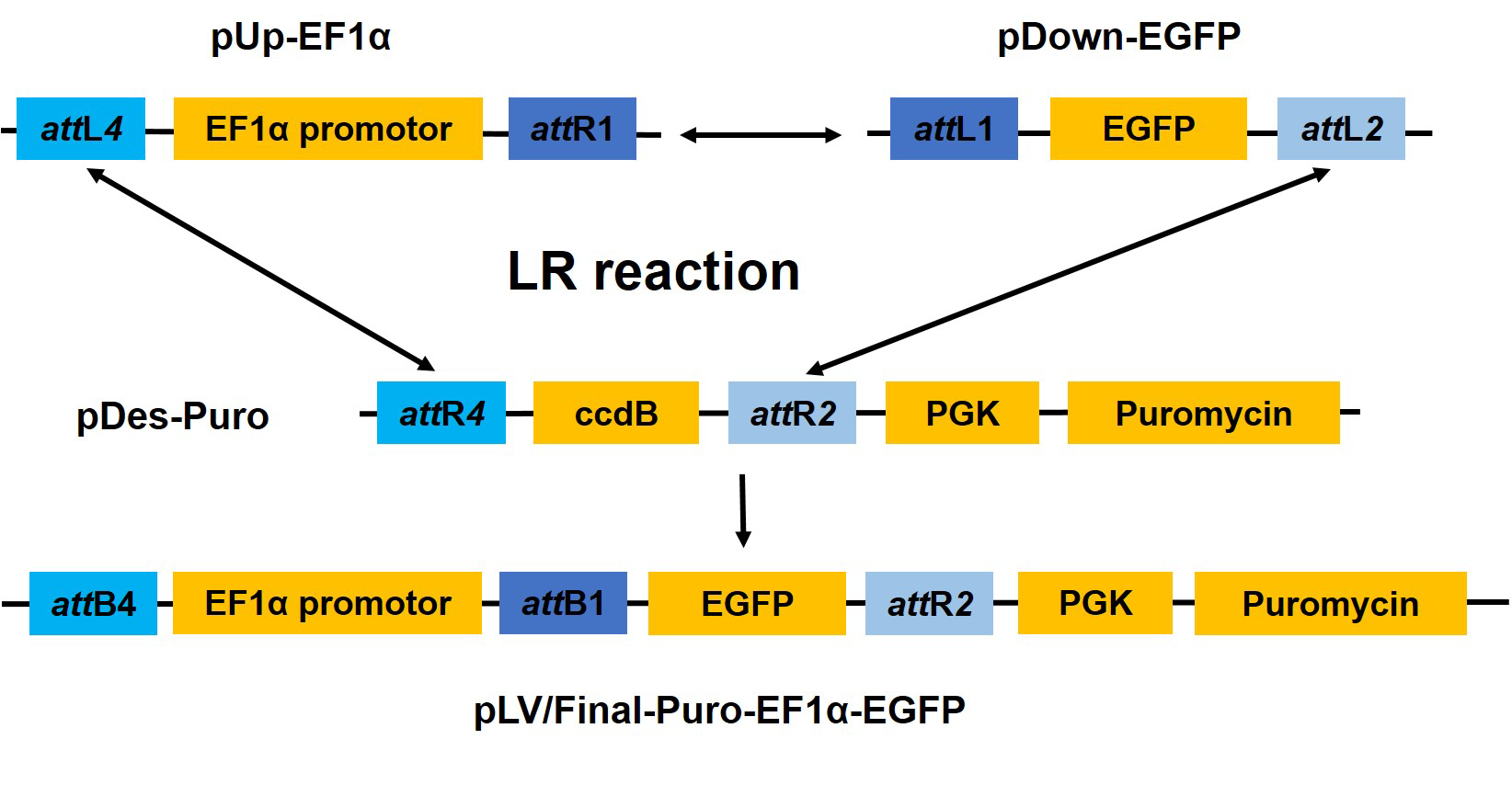

Supplement: Supplementary file 1 — Additional file 1: Figure S1. Schematic diagram of construction of the lentiviral vector pLV/Final-Puro-EF1α-EGFP. The entry vectors, pUp-EF1α and pDown-EGFP were generated and recombined into the pDes-Puro vector utilizing the recognized LR reaction protocol. [file 40659_2020_288_MOESM1_ESM.tif]

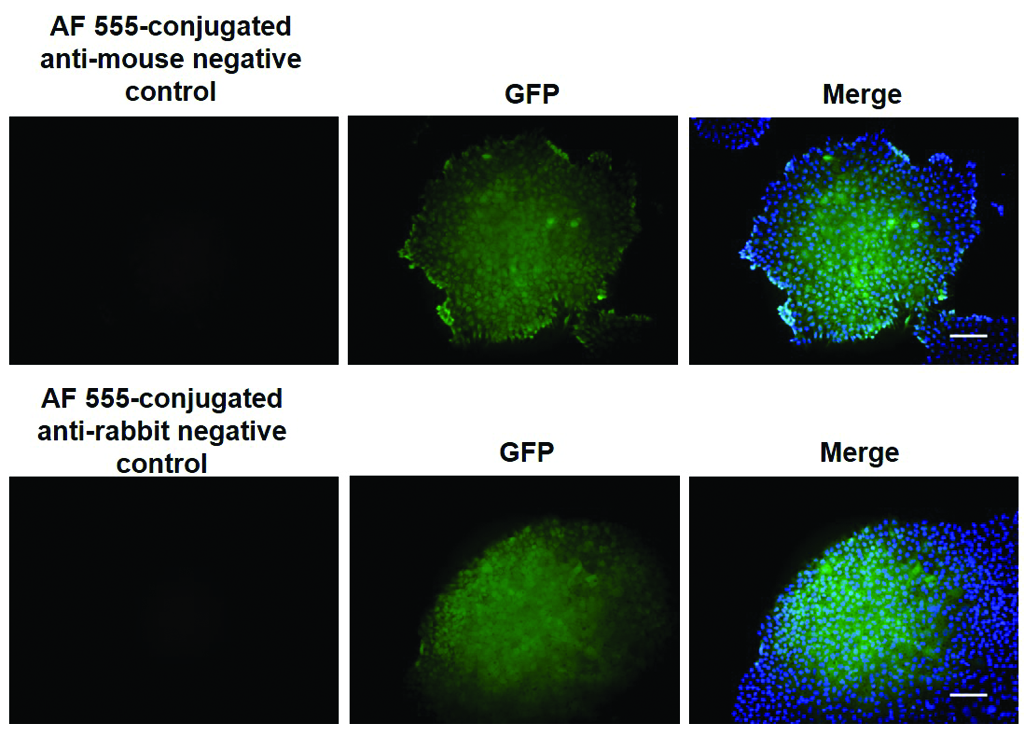

Supplement: Supplementary file 2 — Additional file 2: Figure S2. The negative control of immunocytochemistry analysis on iPSCs. No unspecific immunofluorescence was detected on Alexa Fluor 555-conjugated anti-mouse secondary antibodies (upper panle) and Alexa Fluor 555-conjugated anti-rabbit secondary antibodies (lower panle) without primary antibody. [file 40659_2020_288_MOESM2_ESM.tif]

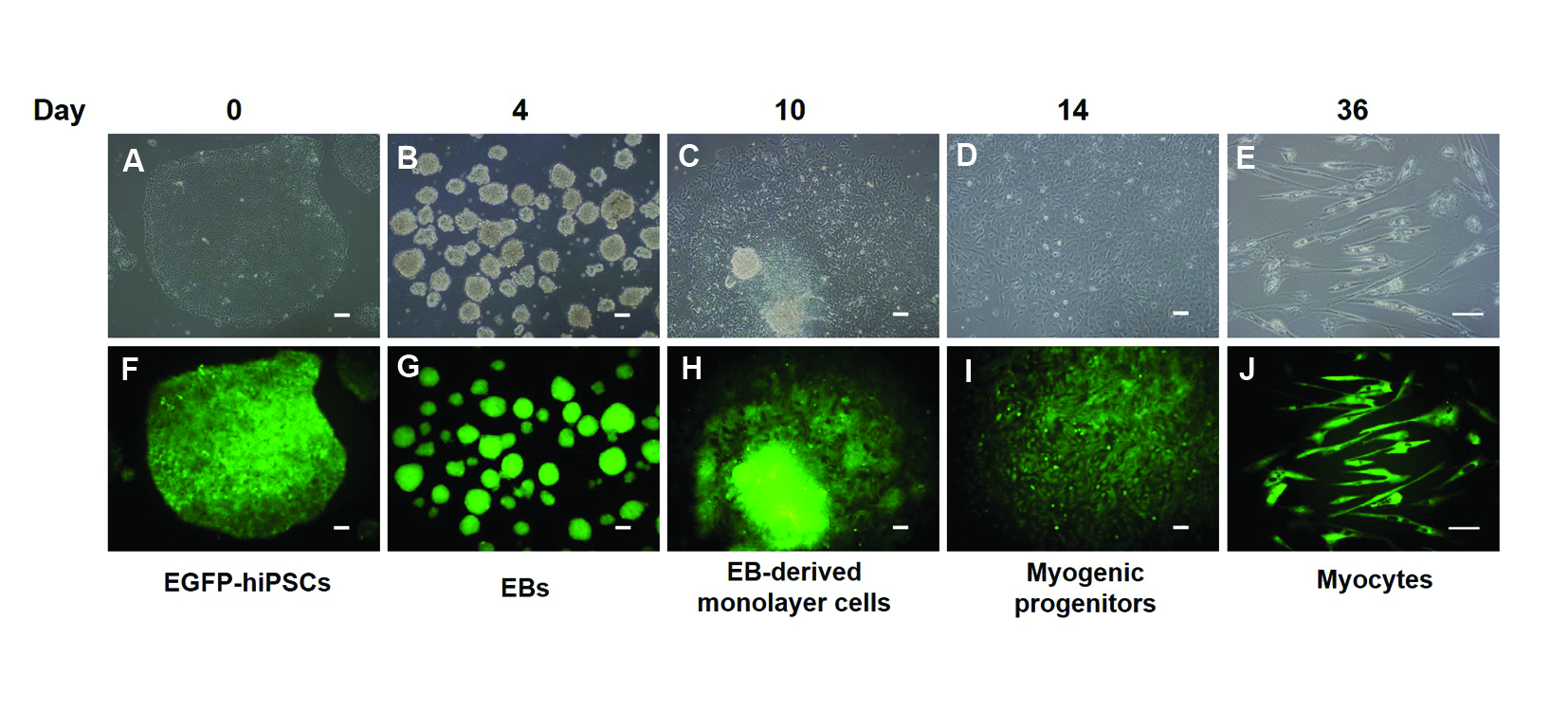

Supplement: Supplementary file 3 — Additional file 3: Figure S3. Representative phase contrast (A-E) and matching GFP fluorescence (F-J) images during myogenic differentiation at several time points. (A, F) EGFP hiPSC colonies at day 0. (B, G) EBs in suspension culture at day 4. (C, H) EB-derived monolayer cells at day 10 after plating EBs on Matrigel-coated plates. (D, I) proliferating monolayer of myogenic progenitors at day 14. (E, J) spindle-shaped myocytes at day 36. Scale bars = 100 μm. [file 40659_2020_288_MOESM3_ESM.tif]

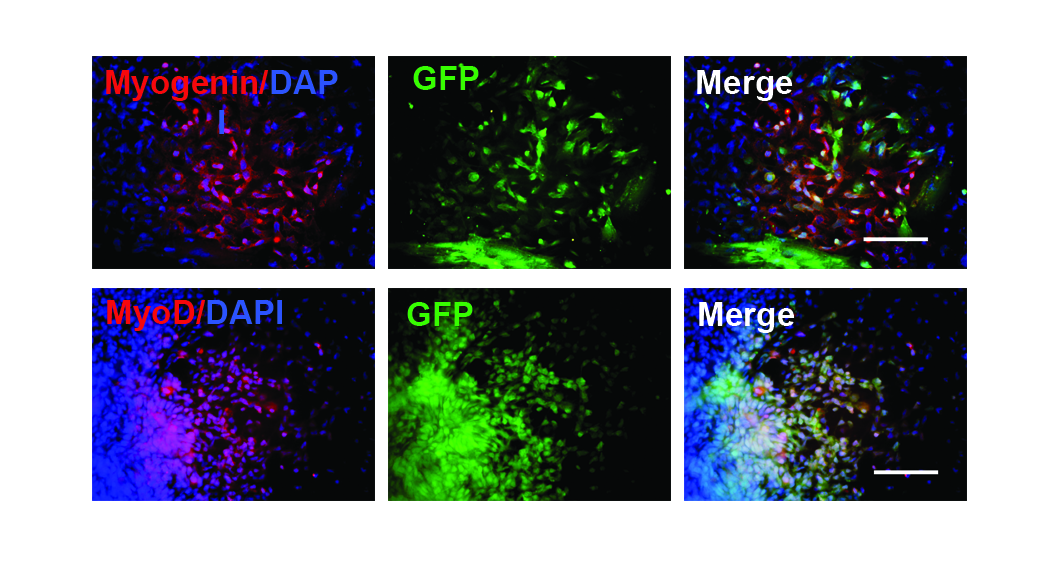

Supplement: Supplementary file 4 — Additional file 4: Figure S4. Immunofluorescence analysis indicated expression of myogenic markers Myogenin and MyoD1 at differentiation day 14. Scale bars = 100 μm. [file 40659_2020_288_MOESM4_ESM.tif]

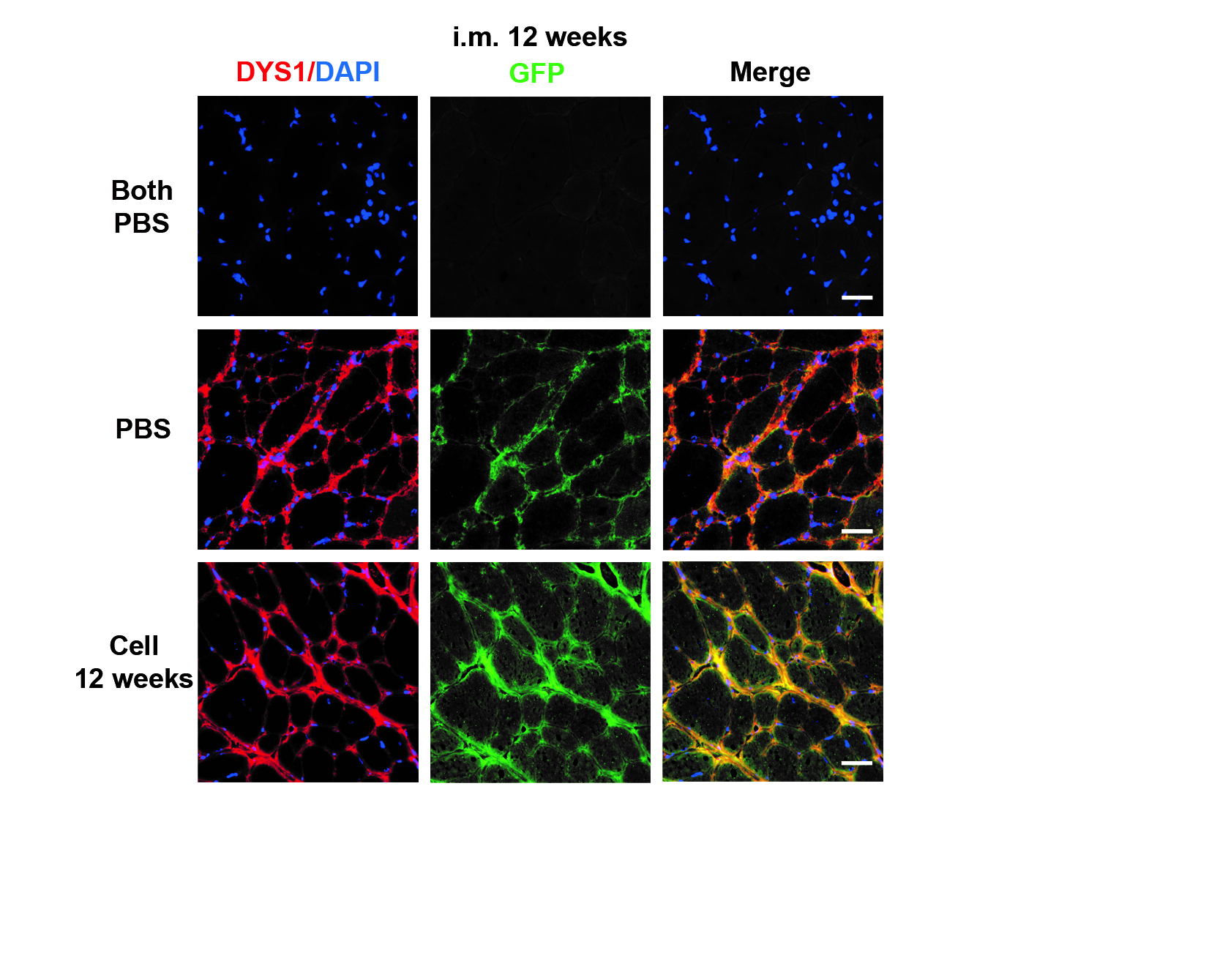

Supplement: Supplementary file 5 — Additional file 5: Figure S5. Immunofluorescence analysis showed no expression of dystrophin (red) and GFP (green) in TA muscles of negative control mdx mice (upper panels), while dystrophin and GFP double expression in PBS-injected right TA muscles (middle panels) and cell-transplanted left TA muscles (lower panels) at 12 weeks after transplantation. Scale bars = 200 μm. [file 40659_2020_288_MOESM5_ESM.tif]

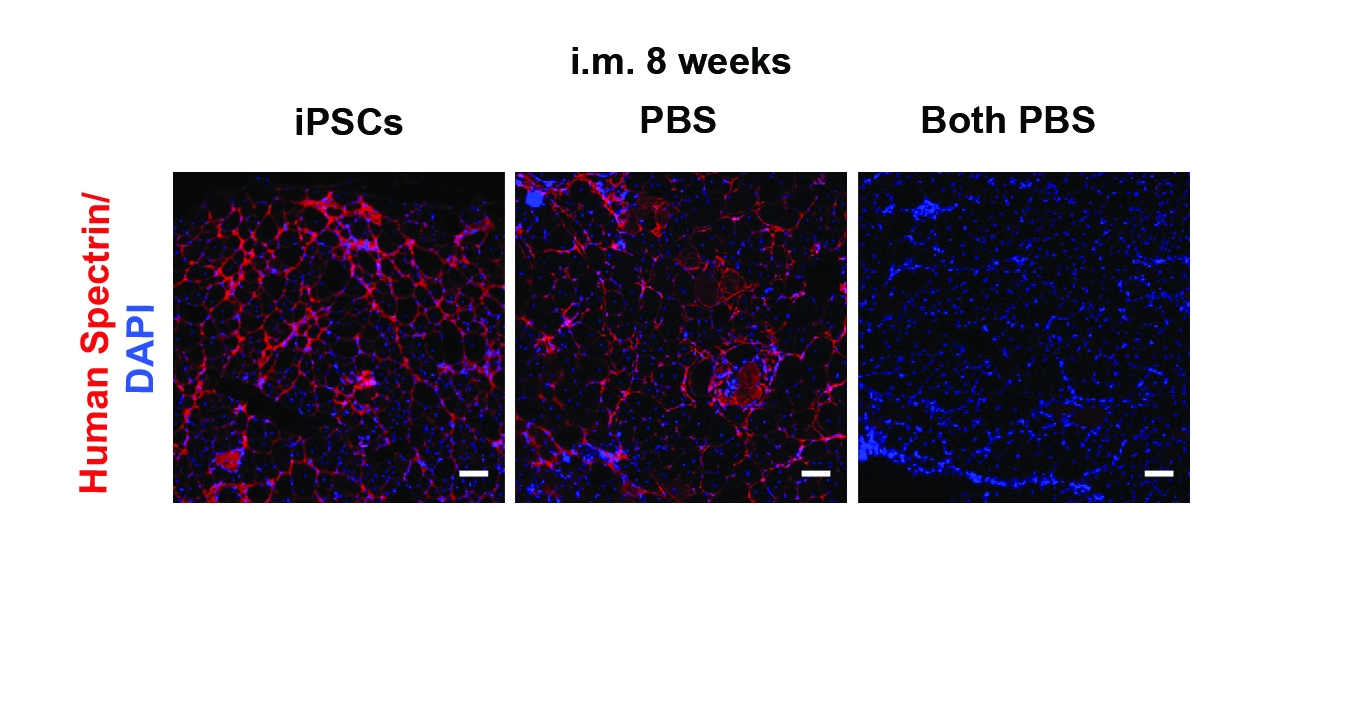

Supplement: Supplementary file 6 — Additional file 6: Figure S6. At 12 weeks after transplantation, immunofluorescence assays showed the expression of human spectrin in the cell-transplanted left TA muscles as well as contralateral muscles. Western blot analysis confirmed the expression of human spectrin. Scale bars = 400 μm. [file 40659_2020_288_MOESM6_ESM.tif]

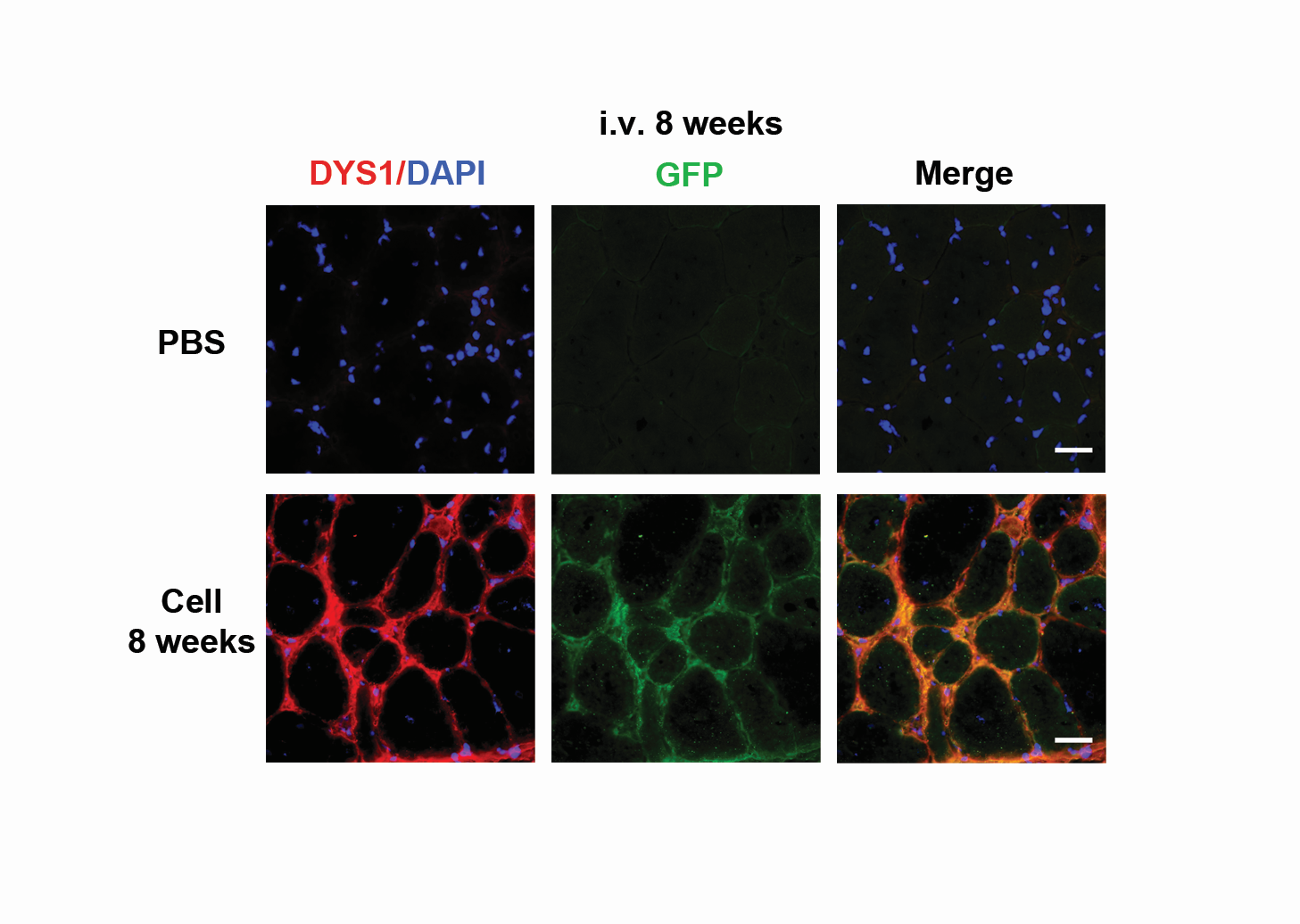

Supplement: Supplementary file 7 — Additional file 7: Figure S7. Immunofluorescence assays showed no dystrophin and GFP expression was observed in the muscles of negative control mdx mice (upper panel), while the expression of dystrophin (red) and GFP (green) in the intravenously-injected TA muscles (lower panel) was detected after 8 weeks of transplantation. Scale bars = 200 μm. [file 40659_2020_288_MOESM7_ESM.tif]

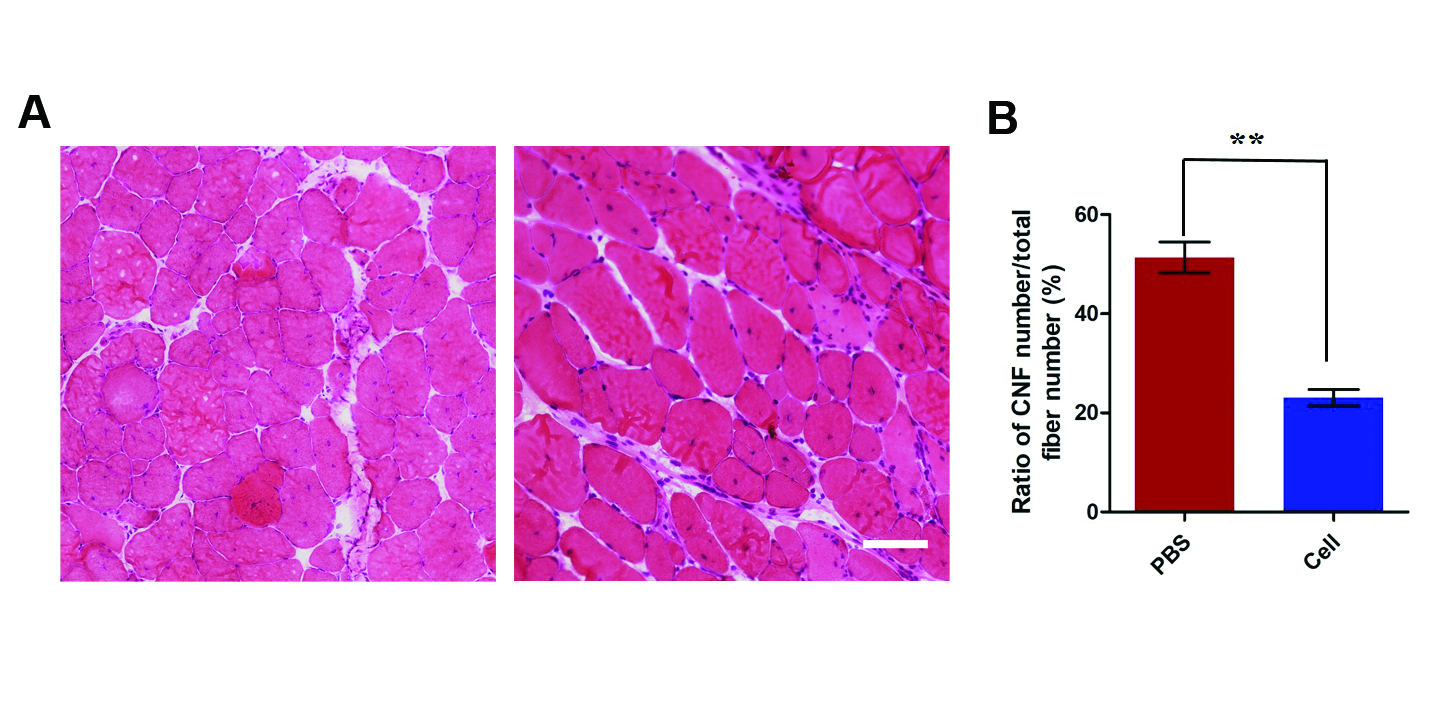

Supplement: Supplementary file 8 — Additional file 8: Figure S8. Systemic transplantation of hiPSC-derived myogenic progenitors without transfecting EGFP reduced the ratio of central nuclei myofibers (CNFs) in mdx mice. (A) H&E staining showed representative images of TA muscles in mdx mice received PBS (left) and cells (right) at 8 weeks after intravenous transplantation. (B) Quantitative analysis indicated the percentage of CNFs for each group at 8 weeks after intravenous transplantation. 5 random sections for each muscle were examined. **P < 0.01, Scale bars = 400 μm. [file 40659_2020_288_MOESM8_ESM.tif]
